# Dexmedetomidine Increases MMP-12 and MBP Concentrations after Coronary Artery Bypass Graft Surgery with Extracorporeal Circulation Anaesthesia without Impacting Cognitive Function: A Randomised Control Trial

**DOI:** 10.3390/ijerph192416512

**Published:** 2022-12-08

**Authors:** Michał Kowalczyk, Anna Panasiuk-Kowalczyk, Adam Stadnik, Małgorzata Guz, Marek Cybulski, Witold Jeleniewicz, Andrzej Stepulak, Magdalena Kwiatosz-Muc

**Affiliations:** 1 1st Department of Anaesthesiology and Intensive Care, Medical University of Lublin, ul. Jaczewskiego 8, 20-954 Lublin, Poland; 2 Department of Cardiac Surgery, Medical University of Lublin, ul. Jaczewskiego 8, 20-954 Lublin, Poland; 3 Department of Biochemistry and Molecular Biology, Medical University of Lublin, ul. Chodźki 1, 20-093 Lublin, Poland

**Keywords:** dexmedetomidine, matrix metalloproteinase-12, myelin basic protein, coronary artery bypass graft surgery, extracorporeal circulation, anaesthesia

## Abstract

Postoperative neurological deficits remain a concern for patients undergoing cardiac surgeries. Even minor injuries can lead to neurocognitive decline (i.e., postoperative cognitive dysfunction). Dexmedetomidine may be beneficial given its reported neuroprotective effect. We aimed to investigate the effects of dexmedetomidine on brain injury during cardiac surgery anaesthesia. This prospective observational study analysed data for 46 patients who underwent coronary artery bypass graft surgery with extracorporeal circulation between August 2018 and March 2019. The patients were divided into two groups: control (CON) with typical anaesthesia and dexmedetomidine (DEX) with dexmedetomidine infusion. Concentrations of the biomarkers matrix metalloproteinase-12 (MMP-12) and myelin basic protein (MBP) were measured preoperatively and at 24 and 72 h postoperatively. Cognitive evaluations were performed preoperatively, at discharge, and 3 months after discharge using Addenbrooke’s Cognitive Examination version III (ACE-III). The primary endpoint was the ACE-III score at discharge. Increased MMP-12 and MBP concentrations were observed in the DEX group 24 and 72 h postoperatively. No significant differences in ACE-III scores were observed between the groups at discharge; however, the values were increased when compared with initial values after 3 months (*p* = 0.000). The current results indicate that the administration of dexmedetomidine as an adjuvant to anaesthesia can increase MMP-12 and MBP levels without effects on neurocognitive outcomes at discharge and 3 months postoperatively.

## 1. Introduction

Neurological outcomes following cardiac surgery with extracorporeal circulation (ECC) remain a significant concern among patients. During ECC, brain injury can occur when small gas, fat emboli, or atherosclerotic plaques obstruct micro-vessels, resulting in regional hypoperfusion [1,2]. While large lesions are immediately noticed following recovery from anaesthesia given obvious clinical symptoms, some minor injuries may be missed. In some cases, slight cognitive decline is the only significant manifestation. This phenomenon, known as postoperative cognitive dysfunction (POCD), is a common cause of disability after cardiac surgery [3,4,5]. Brief neurological assessments conducted during routine practice are often insufficient for identifying the subtle injuries that lead to POCD, highlighting the need for more sensitive indicators of neurocognitive dysfunction [3,4,6,7,8,9]. Indeed, early recognition of the decline in cognitive function allows for immediate psychological support that may improve a patient’s quality of life [10,11].

Alternative methods for identifying neurological injuries include the measurement of biochemical biomarkers such as S100B protein, neuron-specific enolase, glial fibrillary acidic protein, tau protein, myelin basic protein (MBP), matrix metalloproteinase-9 (MMP-9), ubiquitin C-terminal hydroxylase-L1, and neurofilaments. However, the clinical utility of these indicators remains to be determined, and no consensus has been reached [4,12,13,14].

Matrix metalloproteinase-12 (MMP-12) has been identified as a promising new biochemical biomarker of neurological injury [15]. Matrix metalloproteinases (MMPs) are a family of proteolytic enzymes that degrade or modify all components of the extracellular matrix (ECM), playing key roles in inflammation, wound healing, and other pathological processes following neurological injury. MMPs promote blood–brain barrier (BBB) injury, oedema, haemorrhage [16,17,18], and may even trigger brain cell death by disrupting the neurovascular matrix [19,20]. Data suggest that MMPs play a harmful role in stroke development, and several studies have reported a significant upregulation of MMPs following cerebral ischaemia [21,22,23,24]. Moreover, higher MMP-12 levels have been associated with stroke severity and worse neurological outcomes [25]. Research also indicates that MMP-12 can activate other MMPs [26], resulting in the destruction of tight capillary junctions and the basement membrane of the endothelium, BBB damage, increased permeability, and vasogenic brain oedema [15,16,17]. The activation of MMP-12 also induces the degradation of myelin basic protein (MBP) [27] and other substrates such as pro-TNFα, a1-antitrypsin, tissue factor pathway inhibitor, plasminogen, and N-cadherin [27,28,29,30]. Brain ischaemia and reperfusion during cardiac surgery with ECC can promote these pathological processes, highlighting MMP-12 a promising biomarker of brain injury and a potential treatment target [15,24,31].

As mentioned above, MMP-12 can induce the degradation of MBP. MBP is an oligodendroglial cell protein released following brain injury [12]. As noted in other studies, myelin and oligodendrocyte disruption occurs during global ischaemic brain damage [32]; as such, MBP represents another potential biochemical marker of brain injury.

The use of dexmedetomidine in cardiac surgery has been associated with several beneficial effects, including neuroprotection [33,34,35,36]. Dexmedetomidine is a selective alpha-2 adrenoreceptor agonist that induces sedation as well as anxiolytic and analgesic effects without causing respiratory suppression [36,37]. Moreover, dexmedetomidine administration during perioperative care decreases the incidence of delirium and the duration of hospitalisation [34,35]. Although the anti-delirium effect of dexmedetomidine is widely known, few reports have focused on the subtler effects of dexmedetomidine on neurological outcomes, such as neurocognitive function and biochemical markers of brain injury.

In this study, we aimed to determine the effects of dexmedetomidine use during general anaesthesia on neurological outcomes by measuring biochemical markers of brain injury (MMP-12 and MBP) and evaluating neurocognitive outcomes using a validated assessment.

## 2. Materials and Methods

This randomised control trial was approved by the Ethics Committee of the Medical University of Lublin, Poland (approval number: KE-0254/133/2018, Chairperson Prof M. Olajossy, on 24 May 2018). The study was registered at ClinicalTrials.gov (accessed on 15 October 2022) (ID: NCT03585452). Patients were randomly divided into two groups via a simple 1:1 randomisation process using opaque envelopes: a control group (group CON) of patients receiving typical anaesthetic regimens and a dexmedetomidine group (group DEX) of patients receiving an additional dexmedetomidine infusion. Patients were not aware of their group assignments.

**Figure 1:**
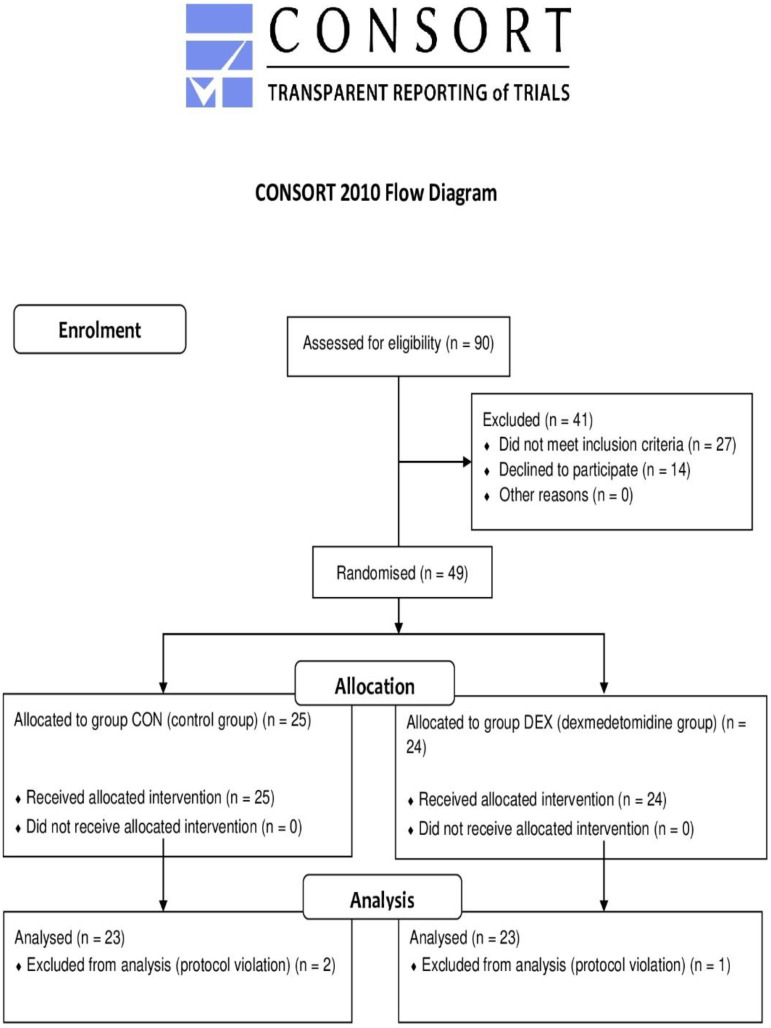
Flow chart of patient selection.

After obtaining written consent, 46 adult patients who qualified for elective coronary artery bypass graft (CABG) surgery with ECC under general anaesthesia were included in the analysis. Inclusion criteria were age > 18 years, elective CABG surgery, an American Society of Anesthesiologists’ Classification (ASA class) of II or III, and an ejection fraction ≥ 40%. Exclusion criteria were ASA class IV or higher, an ejection fraction < 40%, internal carotid or vertebral artery obstruction, severe myocardial infarction, chronic obstructive pulmonary disease, diabetes, and neurological and immunological diseases. Figure 1 shows the flow chart of patient selection in detail.

### 2.1. Anaesthetic and Surgical Procedures

One hour preoperatively, all patients received estazolam (2 mg) per os and morphine (10 mg) subcutaneously as premedication. During anaesthesia monitoring, the following parameters were measured: invasive blood pressure; systolic blood pressure; diastolic blood pressure; mean arterial pressure; heart rate; pulse oximetry; cardiac index (CI), pulmonary capillary wedge pressure, mean pulmonary artery pressure, central vein pressure, systemic vascular resistance index, and pulmonary vascular resistance index via haemodynamic monitoring with a thermodilution pulmonary artery catheter (PAC); end-tidal carbon dioxide (ETCO_2_) to maintain normocapnia (4.0–5.3 kPa); and electroencephalography (EEG) (SedLine, Masimo, Irvine, CA, USA) with the patient state index applied. For the purposes of this study, CI was registered preoperatively, immediately after the surgical procedure, and 24 h thereafter. If patients were stable, the PAC was removed to avoid catheter complications. Moreover, an intravenous (IV) cannula was placed, and an infusion of multi-electrolytic fluid (5–10 mL kg) was started.

Anaesthetic management in group CON was as follows: after pre-oxygenation, a remifentanil bolus (1 µg kg^−1^) was administered, which was followed by an infusion (0.2–0.5 µg kg^−1^ min^−1^), and anaesthesia was induced with etomidate (0.3 mg kg^−1^). Pancuronium (0.1 mg kg^−1^) was subsequently administered for neuromuscular blockade. After appropriate face mask ventilation, the patient was intubated. The maintenance of anaesthesia was continued using a propofol (2–3 mg kg^−1^ h^−1^) infusion. Additional doses of pancuronium (0.03 mg kg ^−1^) were administered as required. The patients were ventilated using an oxygen (O_2_) and air mixture (40% O_2_). The ventilation was guided by ETCO2. A typical CABG procedure with normothermic ECC was performed. Weaning from ECC was performed using dobutamine as inotropic support and nitro-glycerine as a vasodilator, which are both titrated to patients’ dependent doses. Additionally, norepinephrine was used as needed for normotension maintenance under haemodynamic monitoring. The patients were transferred to the intensive care unit (ICU) postoperatively for routine recovery and extubated 4–6 h after ICU admission. In group DEX, the procedure was the same but with an additional dexmedetomidine infusion. After IV cannula placement, a loading dose of 0.5 µg kg^−1^ h^−1^ was administered over 1 h. The dose was reduced to 0.25 µg kg^−1^ h^−1^, and the infusion was continued perioperatively and postoperatively until 200 µg was administered.

In both groups, anaesthetic and opioid doses were adjusted under haemodynamic and EEG sensor control.

After ICU admission, sedation was continued with propofol infusion in group CON and propofol and dexmedetomidine (up to total dose of 200 µg) infusions in group DEX.

### 2.2. Biomarker Measurement

Biomarkers of brain injury (MBP and MMP-12) were assessed preoperatively, before anaesthetic induction, at the end of the surgical procedure, and 24 h and 72 h after the procedure. Venous blood samples were collected using 4.9 mL blood collection tubes. The blood cells were separated via centrifugation for 10 min at 2500× *g*. After centrifugation, serum samples were divided into aliquots and stored at −80 °C. Enzyme-linked immunosorbent assays of MMP-12 and MBP were performed in accordance with the manufacturer’s instructions (R&D Systems, Minneapolis, MN, USA).

### 2.3. Cognitive Function Assessment

Addenbrooke’s Cognitive Examination (ACE) [6] and its most recent version, the ACE-III [7,8,9], are reliable screening tools that can be used to examine the integrity of functioning across five cognitive domains: attention, memory, fluency, language, and visuospatial abilities. In this study, cognitive function was assessed using the Polish version of the ACE-III by researchers who had previously been trained by a clinical psychologist. Assessments were performed at four time points: preoperatively (1 day before surgery), at discharge, 7 days after surgery, and 3 months after discharge. The Polish version of the ACE-III has been validated within the Polish population [38]. The maximum total score is 100 points, with higher scores indicating better cognitive functioning. As noted in previous studies, a cut-off score of 84/100 is used to distinguish cases of dementia [7].

The primary endpoint of the study was determined to be the ACE-III result at discharge.

### 2.4. Statistical Analysis

Statistical analysis was performed using STATISTICA software (StatSoft Polska Sp. z o.o., Kraków, Poland), with the level of statistical significance set at *p* < 0.05. The sample size was calculated using the formula published by Noordzij et al. [39] and was set at a minimum of 21 patients per group. Assumptions for the primary endpoint were a baseline ACE-III score (mean ± standard deviation) of 95.7 ± 3.3 (based on a previous report [7]), an ACE-III score at the time of discharge (mean ± standard deviation) of 86.1 ± 3.3 (assuming 10% deterioration) for group CON, and an ACE-III score at the time of discharge (mean ± standard deviation) of 89 ± 3.3 (assuming 7% deterioration) for group DEX (an alpha of 0.05, and a power goal of 0.9). Descriptive statistics are presented as the mean and standard deviation, median and interquartile range, or number and percentage. All data were checked for normal distribution (using Shapiro–Wilk’s W test) and variance equality when appropriate. To address these assumptions, data were tested using parametric t-tests (two-tailed) for dependent or independent variables as appropriate. Alternatively, they were tested using the non-parametric Wilcoxon test for dependent variables or the Mann–Whitney U-test for independent variables. Furthermore, a difference test between two proportions was used when appropriate. Repeated measures were tested using Friedman’s analysis of variance (ANOVA) and Kendall’s concordance coefficient. Multiple linear regression (MLR) for confounding variables and linear Pearson correlation analyses were performed when appropriate.

## 3. Results

Of 90 patients who were screened for eligibility, 41 were excluded based on patient refusal or not meeting the criteria. Forty-nine patients were randomised, and data were analysed for 46 of these patients. Three patients were excluded from the analysis due to protocol violations, resulting in 23 patients each in the DEX and CON groups.

### 3.1. Patient Characteristics and Perioperative Data

Some between-group differences in patient characteristics and perioperative background factors were observed. Demographic data were similar between the two groups; however, patients in the DEX group required longer surgical and anaesthetic times (*p* = 0.038 and *p* = 0.017, respectively). Patients in the DEX group had lower ejection fractions than those in the CON group (*p* = 0.035). The patients were haemodynamically stable throughout the perioperative period. The confidence interval increased immediately after the surgery and 24 h after the procedure in both the DEX (chi-square ANOVA (N = 23, df = 2) = 35.043; *p* = 0.000) and CON (chi-square ANOVA (N = 23, df = 2) = 44.087; *p* = 0.000) groups. There were no differences in CI between the two groups at any point during the study. In addition, there were no differences between initial plasma concentrations of MBP and MMP-12 or initial results on the ACE-III between the two groups. Detailed characteristics are presented in Table 1.

Both groups were similar in terms of comorbidities; however, patients in the DEX group used diuretics more frequently (*p* = 0.041). The DEX group also had a higher demand for inotrope support (dobutamine) throughout the perioperative period than the CON group (*p* = 0.013). Additionally, patients in the DEX group had received less education than those in the CON group (*p* = 0.019). Detailed data are presented in Table 2.

### 3.2. Biomarker Concentrations

**Figure 2:**
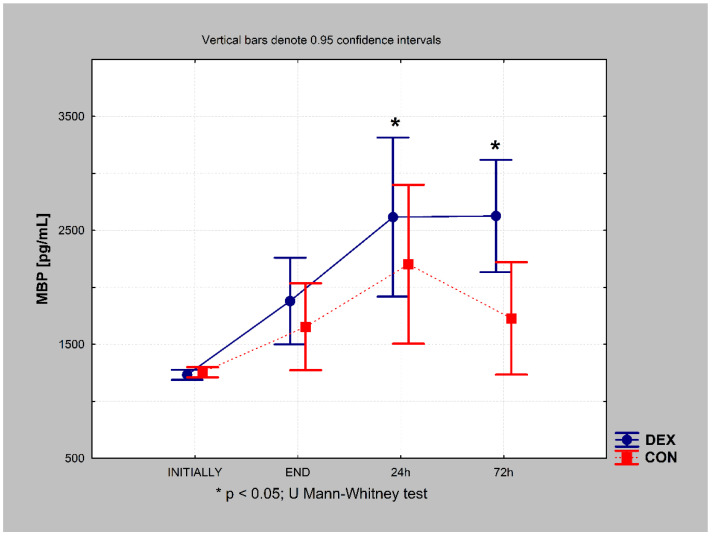
Myelin basic protein (MBP) concentrations in the DEX and CON groups at different time points during the study. CON, control; DEX, dexmedetomidine.

In the DEX group, the MMP-12 concentration increased immediately after surgery, decreased slightly at 24 h, and remained almost the same at 72 h after the procedure (chi-square ANOVA = 25.748; *p* = 0.000). However, the final value (72 h after) was still higher than the preoperative value (*p* = 0.002). In the CON group, the MMP-12 concentration decreased after 24 h (*p* = 0.005) and returned to approximately the preoperative value 72 h postoperatively (chi-square ANOVA = 19.435; *p* = 0.000) (Figure 2).

In the DEX group, postoperative MBP levels increased immediately after surgery, continued to increase 24 h after, and remained increased 72 h after the procedure (chi-square ANOVA = 48.339; *p* = 0.000). In the CON group, postoperative MBP levels increased immediately after surgery, and a further increase was observed 24 h after; however, they decreased 72 h after the procedure. The final value (72 h after) was still higher than the preoperative value in the CON group (chi-square ANOVA = 37.696; *p* = 0.000). 

Cross-over analysis revealed higher MBP concentrations in the DEX group 24 h and 72 h postoperatively (*p* = 0.010 and *p* = 0.006, respectively) (Figure 2).

**Figure 3:**
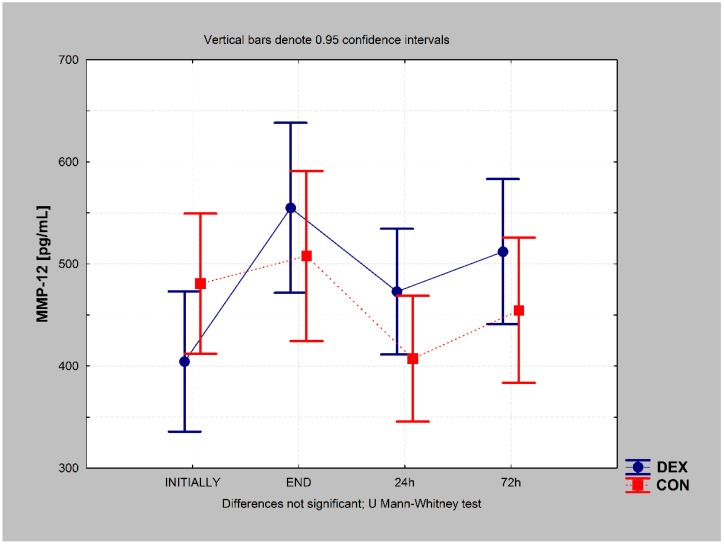
Matrix metalloproteinase-12 (MMP-12) concentrations in the DEX and CON groups at different time points during the study. CON, control; DEX, dexmedetomidine.

There were no significant differences in MMP-12 concentration between the groups at any point during the study (Figure 3). Detailed data related to MMP-12 and MBP concentrations are presented in Table S1.

**Figure 4:**
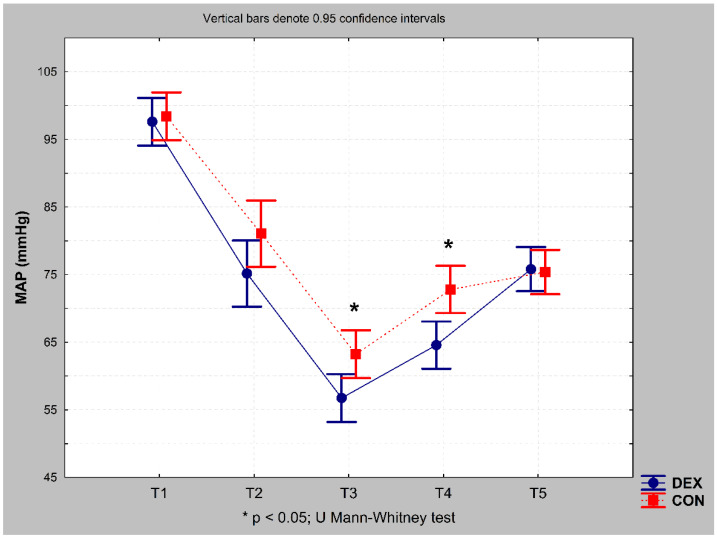
Mean arterial pressure in the DEX and CON groups during the procedure. MAP, mean arterial pressure; T1, initial; T2, 10 min after intubation; T3, 10 min after ECC institution; T4, 10 min after ECC weaning; T5, at the end of the procedure.

During the procedure, mean arterial pressure (MAP) was analysed for the following time points: T1, initial; T2, 10 min after intubation; T3, 10 min after ECC institution; T4, 10 min after ECC weaning; and T5, at the end of the procedure. MAP was significantly lower in the DEX group than in the CON group at T3 (*p* = 0.031) and T4 (0.003) (Figure 4).

### 3.3. Cognitive Function

**Figure 5:**
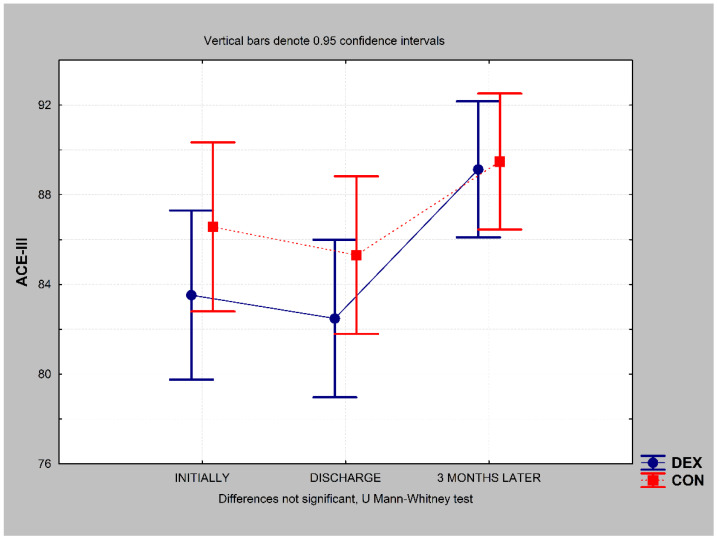
Addenbrooke’s Cognitive Examination version III (ACE-III) results in the DEX and CON groups. CON, control; DEX, dexmedetomidine; Min, minimum; Max, maximum.

Cognitive function as measured using the ACE-III remained the same in patients in the DEX group at the time of discharge (*p* = 0.058) and increased above the preoperative value 3 months postoperatively (*p* = 0.000) (chi-square ANOVA (N = 23, df = 2) = 27.573; *p* = 0.000) (Figure 5). The same outcome was observed in the CON group; cognitive function remained the same at discharge (*p* = 0.323) and significantly increased 3 months later (*p* = 0.000) (chi-square ANOVA (N = 23, df = 2) = 22.024; *p* = 0.000) (Figure 5). There were no between-group differences in ACE-III score at any time point during the study (Figure 5).

### 3.4. Domain-Specific Results for Cognitive Function

#### 3.4.1. Attention

Attention decreased in patients in the DEX group at the time of discharge (*p* = 0.004) and increased slightly above the preoperative value 3 months later (*p* = 0.037) (chi-square ANOVA (N = 23, df = 2) = 17.569; *p* = 0.000). Similar trends were observed in the CON group; attention scores decreased at discharge (*p* = 0.019) and increased to the preoperative value 3 months later (*p* = 0.320) (chi-square ANOVA (N = 23, df = 2) = 13.576; *p* = 0.001).

#### 3.4.2. Memory

In the DEX group, there was no difference in memory scores between the time of discharge and the preoperative value (*p* = 0.134); however, a significant increase was observed 3 months later (*p* = 0.001) (chi-square ANOVA (N = 23, df = 2) = 12.819, *p* = 0.001). There were no differences in memory scores in the CON group at any time point during the study (chi-square ANOVA (N = 23, df = 2) = 2337; *p* = 0.311).

#### 3.4.3. Fluency

In the DEX group, there were no changes in fluency at the time of discharge (*p* = 0.146), and a significant increase was observed 3 months postoperatively when compared with the preoperative value (*p* = 0.000) (chi-square ANOVA (N = 23, df = 2) = 20,425, *p* = 0.000). The same trend was observed in the CON group, with no change at discharge (*p* = 0.387) and a significant increase 3 months later (*p* = 0.002) (chi-square ANOVA (N = 23, df = 2) = 15,687; *p* = 0.000).

#### 3.4.4. Language

Initial and discharge language scores were similar in the DEX group (*p* = 0.065), although values were lower 3 months postoperatively (*p* = 0.041) (chi-square ANOVA (N = 23, df = 2) = 8787; *p* = 0.012). In the CON group, there were no differences at any time point during the study (*p* = 0.859 at the time of discharge and *p* = 0.799 3 months postoperatively) (chi-square ANOVA (N = 23, df = 2) = 0.057; *p* = 0.972).

#### 3.4.5. Visuospatial Abilities

Despite no changes in visuospatial ability at the time of discharge based on repeated-measures ANOVA for the DEX group (chi-square ANOVA (N = 23, df = 2) = 4.275; *p* = 0.118), the values at 3 months postoperatively were higher than the preoperative values (Wilcoxon test, *p* = 0.035). In the CON group, there were no differences at discharge or 3 months postoperatively (chi-square ANOVA (N = 23, df = 2) = 2.164; *p* = 0.339).

Using an ACE-III cut-off score of 84, we observed no significant differences in the dementia rate between the two groups at any time point. The detailed data are presented in Table 3. The detailed ACE-III results are presented in Table S2.

### 3.5. Regression Analysis

MLR analysis was performed to evaluate the possible influence of variables that differed significantly between the DEX and groups (EF, surgical time, anaesthetic time, and dobutamine dosage) on outcome variables (MMP-12, MBP, and ACE-III). Assumptions were as follows: EF, surgical time, anaesthetic time, and dobutamine dosage per kg of body weight were considered independent variables (predictors, confounding variables), while the outcome variables of MMP-12, MBP, and ACE-III were considered dependent variables. Surgical time was strongly correlated with anaesthetic time; therefore, anaesthetic time was introduced into the MLR model, which revealed no significant influence of EF, anaesthetic time, and dobutamine dosage on MBP levels (preoperatively, before anaesthetic induction, at the end of the surgical procedure, and 24 h and 72 h after the procedure) in the DEX or CON group. We also observed no significant influence of EF, anaesthetic time, or dobutamine dosage on MMP-12 levels (preoperatively, before anaesthetic induction, at the end of the surgical procedure, and 24 h and 72 h after the procedure) in the DEX group. However, anaesthetic time significantly (*p* < 0.05, b > 0) influenced MMP-12 levels (preoperatively, before anaesthetic induction, at the end of the surgical procedure, and 24 h and 72 h after the procedure) in the CON group. Longer anaesthetic time correlated with higher MMP-12 levels at all study points. Additionally, we observed no significant influence of EF, anaesthetic time, or dobutamine dosage on ACE-III scores (initially, discharge, and 3 months later) in the DEX group. However, dobutamine dosage significantly (*p* = 0.018, b < 0) influenced ACE-III scores 3 months after discharge in the CON group. Higher dobutamine dosage correlated with lower ACE-III scores 3 months after discharge.

## 4. Discussion

Our study revealed that patients anaesthetised with dexmedetomidine as an adjuvant for CABG procedures had increased MMP-12 and MBP concentrations perioperatively. The literature reveals that higher MMP-12 levels have been associated with worse neurological outcome [25]. MMP-12 upregulation has also been associated with brain ischemia in animal models, with post-ischemic induction higher for MMP-12 than for any other MMPs [40,41]. Other studies have demonstrated that MMP-12 induces brain injury by damaging the BBB after focal cerebral ischemia, while MMP-12 knockdown attenuates this effect [15,42]. Recent reports have revealed that MMP-12 suppression can improve neurological outcomes in animal models of brain ischemia, making MMP-12 a promising therapeutic target [24,31]. The patterns of change in MMP-12 and MBP concentrations observed in the present study are in accordance with those reported in previous studies, suggesting that the activation of MMP-12 induces MBP degradation [27]. Furthermore, animal models have demonstrated that global cerebral ischaemia induces neuronal myelin loss, resulting in neurodegeneration [32]. Our data suggest that at each study time point, higher MMP-12 concentrations were associated with higher MBP levels. MBP is an oligodendroglial cell protein; hence, an increase in the level of this biomarker suggests brain injury, which may explain the higher MBP concentrations 24 and 72 h postoperatively in the DEX group.

Our data also indicated that MAP decreased during ECC and after ECC weaning in the DEX group when compared with that in the CON group. This is in the line with the results of a recent meta-analysis reporting lower blood pressure and risk of hypotension in patients receiving dexmedetomidine before intubation [43]. Patients undergoing ECC for CABG procedures with dexmedetomidine have also exhibited a lower hemodynamic response to surgical stress during and after ECC [44,45]. Lower blood pressure may be a risk factor of decreases in local or global brain perfusion, which may increase MMP-12 and MBP levels, although further studies are required to examine the validity of this hypothesis.

In addition to blunting the haemodynamic response, dexmedetomidine use in cardiac surgery settings has been shown to exert antiarrhythmic effects [46,47], reduce post-surgical myocardial injury, and shorten the duration of mechanical ventilation and the ICU stay [48,49]. Dexmedetomidine also exerts neuroprotective effects, with studies reporting decreased neurological injury after cardiac surgery [33], lowered delirium incidents [35,45], and even reductions in the mortality rate [34,50]. Furthermore, a recent meta-analysis identified dexmedetomidine use as a protective factor for postoperative cognitive function after general anaesthesia in patients undergoing non-cardiac surgery [51]. Other studies have reported that dexmedetomidine administration during anaesthesia can decrease the incidence of POCD in older adults undergoing non-cardiac surgery [52,53] and CABG without adverse effects on circulatory function [54].

In this study, we observed no significant differences in ACE-III results between the study groups at any time point, although ACE-III scores surprisingly increased 3 months after discharge. According to the ACE-III results, there were also no significant differences in dementia rates between the groups at any time point in the study. Furthermore, the dementia rate decreased 3 months after discharge in both groups. These improvements in neurocognitive function 3 months after surgery may reflect the effect of proper heart revascularisation, which may have improved exercise tolerance and perfusion, including that of the brain. This in turn may have resulted in better cognitive functioning and outcomes. In the literature, POCD after cardiac surgery has been reported in 30–80% of cases a few weeks postoperatively and in 10–60% of cases after 3–6 months, depending on the definition and time of assessment [5]. One study reported a cognitive decline of 53% at hospital discharge, 36% after 6 weeks, and 24% after 6 months [55]. Another reported a cognitive deterioration of 26% 1 week after non-cardiac surgery and 10% after 3 months [56]. Although we observed no deterioration of cognitive function in our study, neurocognitive testing is essential in clinical settings. The quick recognition of cognitive decline enables the rapid initiation of psychological support that can improve the patient’s quality of life [57,58]. Indeed, persistent POCD has been shown to worsen quality of life 1 year postoperatively [10] and negatively affect patients’ careers 5 years postoperatively [59]. Our data do not support a reduced incidence of POCD after cardiac surgery with dexmedetomidine use; nevertheless, the well-established benefits mentioned above fully justify using dexmedetomidine more widely in cardiac surgery settings.

In this study, we used the most recent version of the ACE [7]. The diagnostic value of the ACE-III is as good as that of other standardised screening instruments, such as the Rowland Universal Dementia Assessment Scale, Montreal Cognitive Assessment, memory impairment screen, and Mini-Mental State Examination (MMSE) [8,9]. The ACE-III has been translated and validated within Polish population. Its higher sensitivity and specificity than the MMSE suggest that the ACE-III should be recommended for use as a cognitive screening tool [38].

This study had some limitations, including the observed differences in patient characteristics and perioperative background data between the study groups. MLR analysis revealed a significant influence of anaesthetic time on MMP-12 results in the CON group, with longer anaesthetic time exhibiting an association with higher MMP-12 values at all study points. In addition, higher dobutamine dosage correlated with lower ACE-III scores 3 months after discharge in the CON group. Despite these correlations, the effects on study outcomes appears to have been insignificant, although further studies are required to clarify these issues.

## 5. Conclusions

Our data suggest that dexmedetomidine used as an adjuvant to general anaesthesia during elective CABG procedures with ECC increases the concentrations of biomarkers such as MMP-12 and MBP. However, dexmedetomidine use does not appear to impact neurocognitive outcomes based on ACE-III results.

## Figures and Tables

**Table 1:** Patient characteristics.

	Group DEX (n = 23)	Group CON (n = 23)	*p*-Value
Age (years)	67 (10)	66 (5)	0.854
Weight (kg)	76 (19)	83 (16)	0.151
Height (cm)	167 (10)	170 (9)	0.282
EF (%)	52 (45–60)	60 (50–61)	**0.035**
Surgical time (min)	193 (47)	168 (31)	**0.038**
Anaesthetic time (min)	248 (49)	218 (32)	**0.017**
Clamping time (min)	49 (9)	42 (12)	0.166
ECC time (min)	79 (29)	67 (18)	0.078
MMP-12 initial value (pg/mL)	388 (303–474)	433 (352–563)	0.267
MBP initial value (pg/mL)	1223 (1179–1302)	1243 (1173–1339)	0.637
ACE-III initially (score 0–100)	83 (74–92)	88 (81–92)	0.323
CI initial value	1.9 (1.6–2.1)	1.9 (1.7–2.1)	0.482
CI after surgery	2.2 (2.0–2.4)	2.4 (2.2–2.7)	0.057
CI 24 h after surgery	3.1 (2.8–3.3)	3.2 (2.8–3.4)	0.792

Data are expressed as the mean (standard deviation) or the median (interquartile range). ACE-III, Addenbrooke’s Cognitive Examination version III; CI, cardiac index; CON, control; DEX, dexmedetomidine; EF, ejection fraction; ECC, extracorporeal circulation; MBP, myelin basic protein; MMP-12, matrix metalloproteinase-12. Significant results are presented in bold.

**Table 2:** Perioperative background data.

	Group DEX (n = 23)	Group CON (n = 23)	*p*-Value
Comorbidities, n (%)			
Myocardial infarction history	15 (65)	10 (43)	0.141
Heart muscle contractility disorders	11 (48)	14 (61)	0.381
Atrial fibrillation	4 (17)	3 (13)	0.706
Congestive heart failure	2 (9)	1 (4)	0.495
Hypertension	21 (91)	18 (78)	0.230
Thyroid disease	3 (13)	4 (17)	0.706
Preoperative medications, n (%)			
β-Blockers	19 (83)	20 (87)	0.706
Calcium channel blockers	12 (52)	8 (35)	0.251
ACE inhibitors	15 (65)	9 (39)	0.085
Angiotensin II receptor blocker	5 (22)	8 (35)	0.334
Diuretics	12 (52)	5 (22)	**0.041**
Statins	23 (100)	22 (96)	0.338
Alcohol intake, n (%)			
Occasional	12 (52)	14 (63)	0.455
None	11 (48)	9 (39)	0.541
Years of education, median (IQR)	10 (8–11)	12 (10–14)	**0.029**
Intraoperative opioids (remifentanil)			
Total dose (µg), mean (SD)	4892 (1665)	5230 (2018)	0.886
Total dose/kg (µg/kg), mean (SD)	63.8 (17.4)	62.1 (19.2)	0.748
ICU length of stay (days), median (IQR)	2 (2–3)	3 (2–3)	0.138
Intubation time (hours), median (IQR)	6.1 (4.9–7.0)	7.0 (5.0–7.8)	0.195
Re-exploration for bleeding, n (%)	2 (9)	1 (4)	0.495
Inotrope (dobutamine) dosage			
Total (mg), median (IQR)	305 (208–450)	133 (30–390)	**0.013**
Total/kg (mg/kg), median (IQR)	4.3 (2.8–7.6)	2.0 (0.4–3.9)	**0.005**
Vasoconstrictor (norepinephrine) use, n (%)	4 (17)	2 (9)	0.424
Delirium, n (%)	2 (9)	5 (22)	0.230

Data are expressed as the n (%), median (IQR), or mean (SD). ACE, angiotensin-converting enzyme; CON, control; DEX, dexmedetomidine; ICU, intensive care unit; IQR, interquartile range; SD, standard deviation. Significant results are presented in bold.

**Table 3:** Dementia rate at different time points during the study.

	Group DEX (n = 23)	Group CON (n = 23)	*p*-Value
Initially	12 (52)	8 (35)	0.251
Discharge time	10 (44)	9 (39)	0.732
3 months after discharge	6 (26)	5 (22)	0.752

Data are expressed as the n (%). CON, control; DEX, dexmedetomidine.

## Data Availability

The datasets generated during and/or analysed during the current study are available from the corresponding author on reasonable request.

## Supplementary Materials

The following supporting information can be downloaded at: https://www.mdpi.com/article/10.3390/ijerph192416512/s1, Table S1: MMP-12 and MBP concentrations in the DEX and CON groups; Table S2: Addenbrooke’s Cognitive Examination version III in the DEX and CON groups.
