# Supplementary material for: Dexmedetomidine Increases MMP-12 and MBP Concentrations after Coronary Artery Bypass Graft Surgery with Extracorporeal Circulation Anaesthesia without Impacting Cognitive Function: A Randomised Control Trial
Source: Int J Environ Res Public Health. 2022 Dec 8;19(24):16512. doi: 10.3390/ijerph192416512 (PMC9778911; doi:10.3390/ijerph192416512)
Supplement: Supplementary file 1 [file ijerph-19-16512-s001.zip › Table S1.pdf]

Table S1. MMP-12 and MBP concentrations in the DEX and CON groups

	**DEX** (n = 23)	**CON** (n = 23)	p-value
	Median (IQR)	Median (IQR)	
MMP-12 initially (pg/mL)	388 (303–474)	433 (352–563)	0.272
MMP-12 end of surgery (pg/mL)	555 (388–721)	437 (381–568)	0.219
MMP-12 24 h after surgery (pg/mL)	475 (391–535)	376 (300–497)	0.075
MMP-12 72 h after surgery (pg/mL)	484 (421–599)	379 (338–551)	0.068
MBP initially (pg/mL)	1223 (1179–1302)	1243 (1173–1339)	0.645
MBP end of surgery (pg/mL)	1587 (1348–1917)	1488 (1344–1709)	0.345
MBP 24 h after surgery (pg/mL)	2178 (1772–3206)	1869 (1511–2142)	**0.010**
MBP 72 h after surgery (pg/mL)	2239 (1636–3446)	1519 (1293–1780)	**0.006**

All values were obtained using the Mann-Whitney U test.

CON, control; DEX, dexmedetomidine; IQR: interquartile range; MBP, myelin basic protein; MMP-12, matrix metalloproteinase-12.
