# Supplementary material for: Dexmedetomidine Increases MMP-12 and MBP Concentrations after Coronary Artery Bypass Graft Surgery with Extracorporeal Circulation Anaesthesia without Impacting Cognitive Function: A Randomised Control Trial
Source: Int J Environ Res Public Health. 2022 Dec 8;19(24):16512. doi: 10.3390/ijerph192416512 (PMC9778911; doi:10.3390/ijerph192416512)
Supplement: Supplementary file 1 [file ijerph-19-16512-s001.zip › Table S2.pdf]

Table S2. Addenbrooke's Cognitive Examination version III in the DEX and CON groups

	DEX (n = 23)	CON (n = 23)	p-value
	Median (IQR)	Median (IQR)	
ACE-III initially (0–100)	83(74–92)	88 (81–92)	0.327
ACE-III at discharge (0–00)	84 (78–90)	88 (80–91)	0.350
ACE-III 3 months later (0–100)	86 (83–95)	92 (84–95)	0.843
Attention initially (0–18)	18 (16–18)	18 (17–18)	0.838
Attention at discharge (0–18)	16 (15–17)	16 (15–18)	0.733
Attention 3 months later (0–18)	18 (17–18)	18 (17–18)	0.956
Memory initially (0–26)	19 (18–24)	20 (19–25)	0.208
Memory at discharge (0–26)	20 (18–24)	21 (19–25)	0.282
Memory 3 months later (0–26)	23 (20–25)	24 (19–25)	0.553
Fluency initially (0–14)	10 (7–11)	10 (7–11)	0.843
Fluency at discharge (0–14)	9 (7—10)	9 (8–11)	0.475
Fluency 3 months later (0–14)	11 (9–14)	11 (9–12)	0.709
Language initially (0–26)	26 (23–26)	26 (24–26)	0.792
Language at discharge (0–26)	25 (24–26)	26 (24–26)	0.297
Language 3 months later (0–26)	25 (22–26)	26 (24–26)	0.150
Visuospatial abilities initially (0–16)	14 (11–15)	14 (13–15)	0.240
Visuospatial abilities at discharge (0–16)	14 (12–15)	14 (13–15)	0.538
Visuospatial abilities 3 months later (0–16)	15 (12–15)	14 (13–15)	0.132

All values were obtained using the Mann-Whitney U test.

ACE-III, Addenbrooke's Cognitive Examination version III; CON, control; DEX, dexmedetomidine;

IQR: interquartile range.
